# Proteomic Investigation of Molecular Mechanisms in Response to PEG-Induced Drought Stress in Soybean Roots

**DOI:** 10.3390/plants11091173

**Published:** 2022-04-26

**Authors:** Ying Zhou, Huiying Li, Haoran Chen, Xiaoqin Yang, Tingting Yu, Yushuang Wang, Yujue Wang, Keting Jiang, Yan Wang, Zhanyu Chen, Xiyan Cui

**Affiliations:** 1 College of Life Sciences, Jilin Agricultural University, Changchun 130118, China; yzhou@jlau.edu.cn (Y.Z.); lihuiying@mails.jlau.edu.cn (H.L.); chr15375437721@163.com (H.C.); 20211235@mails.jlau.edu.cn (X.Y.); wangyujue@mails.jlau.edu.cn (Y.W.); jiangketing0620@163.com (K.J.); wangyansk@jlau.edu.cn (Y.W.); 2 Key Laboratory of Molecular Epigenetics of the Ministry of Education, Northeast Normal University, Changchun 130024, China; yutt945@nenu.edu.cn; 3 College of Agronomy, Jilin Agricultural University, Changchun 130118, China; 20200026@mails.jlau.edu.cn

**Keywords:** soybean root, glutathione, drought stress, physiological response, proteomics, antioxidant, enrichment analysis, tandem mass tag, gene ontology

## Abstract

Roots are generally the critical drought sensors, but little is known about their molecular response to drought stress. We used the drought-tolerant soybean variety ‘Jiyu 47’ to investigate the differentially expressed proteins (DEPs) in soybean roots during the seedling stage based on the tandem mass tag (TMT) proteomics analysis. Various expression patterns were observed in a total of six physiological parameters. A total of 468 DEPs (144 up-regulated and 324 down-regulated) among a total of 8687 proteins were identified in response to drought stress in 24 h. The expression of DEPs was further validated based on quantitative real-time PCR of a total of five genes (i.e., *GmGSH*, *GmGST1*, *GmGST2* k *GmCAT*, and *Gm6PGD*) involved in the glutathione biosynthesis. Results of enrichment analyses revealed a coordinated expression pattern of proteins involved in various cellular metabolisms responding to drought stress in soybean roots. Our results showed that drought stress caused significant alterations in the expression of proteins involved in several metabolic pathways in soybean roots, including carbohydrate metabolism, metabolism of the osmotic regulation substances, and antioxidant defense system (i.e., the glutathione metabolism). Increased production of reduced glutathione (GSH) enhanced the prevention of the damage caused by reactive oxygen species and the tolerance of the abiotic stress. The glutathione metabolism played a key role in modifying the antioxidant defense system in response to drought stress in soybean roots. Our proteomic study suggested that the soybean plants responded to drought stress by coordinating their protein expression during the vegetative stage, providing novel insights into the molecular mechanisms regulating the response to abiotic stress in plants.

## 1. Introduction

The growth and development of crop plants are generally inhibited by both abiotic and biotic stresses, e.g., drought, salt, cold, and diseases [1,2,3]. Drought has become one of the most important issues in the study of the effects of climate change on agricultural productivity worldwide [4,5,6]. A wide range of morphological, physiological, and molecular variations are caused by drought stress in various types of organs and tissues of plants [2,7]. Subsequently, over the course of long-term evolution and natural selection, plants have developed a variety of strategies to cope with drought stress, including drought escape via developmental plasticity and drought tolerance via enhanced osmotic adjustment, water absorption, antioxidant capacity, and stomatal adjustment [8,9]. For example, studies have shown that the adaptation to drought conditions is regulated by the activation of a number of interconnected molecular mechanisms underlying several metabolic processes, including stress-signal reception, signal transduction, gene and protein expression, and cellular metabolic reaction [10,11,12].

As the most widely distributed legume plants worldwide, soybeans are a low-cost source of proteins and vegetable oil for humans. However, the increasing demand of soybean production and overall performance of soybean have been significantly limited and even threatened by drought stress, while drought tolerance is generally a challenging quantitative attribute to investigate. Studies have shown that drought stress has a significant impact on the physiological processes and metabolisms, while several genes involved in the response to water deficiency stress have been identified in soybean [13,14]. As a result, it is practically important to further explore the molecular mechanisms in response to drought stress in soybean at transcriptional, translational, and metabolic levels. To date, a variety of transcriptomic and metabolomic investigations have been conducted to explore the molecular processes of plant response to water deprivation [15,16]. Over the past several years, proteomic studies have identified a large number of proteins involved in several critical metabolic processes, including the glucose and amino acid metabolisms, the redox homeostasis, the stress response, the photosynthesis, the signal transmission, and the protein processing [17,18,19], demonstrating evidently the significance of proteomics in deciphering the molecular mechanisms regulating various biological processes in plants. Briefly, proteomics is the comprehensive study of the structural and functional features of all proteins in an organism. It is naturally expected that the application of proteomics analysis plays a critical role in the exploration of the complicated biological systems, such as the plant response to abiotic stress [20]. For example, the proteomics has been recognized as one of the most commonly utilized techniques for investigating the relationships among the functions of genes and related molecular mechanisms [20].

Studies have shown that under drought stress, crop plants generally use a variety of antioxidant enzymes, e.g., superoxide dismutase (SOD), catalase (CAT), ascorbate peroxidase (APX), and glutathione reductase (GR), as well as antioxidants, e.g., glutathione (GSH), to alleviate the oxidative stress induced by droughts [21]. In particular, as a type of important signaling molecule, the GSH is involved in various types of metabolic pathways to regulate the coordinated interactions among seed germination, stomatal closure, and drought resistance [22]. Furthermore, glutathione peroxidase (GPX) plays an important role in reactive oxygen species (ROS) scavenging by catalyzing the reduction in H_2_O_2_ and other organic hydroperoxides to protect plant cells from the damage caused by oxidative stress, ultimately increasing drought resistance [23]. Additionally, studies have shown that the overexpression of maize sulfite oxidase encoded by *ZmSO* enhanced drought tolerance in transgenic tobacco plants by possibly regulating the expression of genes involved in stomatal regulation, GSH-dependent antioxidant system, and sulfur metabolism [24]. The molecular response involved in these metabolic pathways to drought stress in soybean roots remains uncharacterized at the translational level based on the tandem mass tag (TMT) methodology.

Studies have shown that the common set of proteins involved in the reactive oxygen species (ROS) system, the carbon metabolism, the photosynthesis, the signal transduction, and the amino acid metabolism have been revealed in diverse stress responses in soybeans [25,26,27]. It is speculated that these proteins are potentially involved in the genetic and molecular regulations of stress response in soybeans [28]. Furthermore, studies have shown that the variations in protein abundance are the most sensitive response to drought stress in soybean roots [29]. As one of the main locations responsible for receiving stress signals, the root systems initiate a series of alterations in gene expression in response to drought stress, while the water absorption in roots is the key to triggering drought resistance [29]. Moreover, studies have shown that the expressions of a large number of proteins involved in a variety of cellular functions, such as the carbohydrate and nitrogen metabolisms, the cell wall modification, the signal transduction, the cellular defense, and the programmed cell death, are largely altered in soybean roots but are restored at the seedling stage under drought stress [30]. For example, the proteomic analysis of soybean roots under drought conditions has revealed that two key enzymes involved in carbohydrate metabolism, i.e., the UDP glucose pyrophosphorylase and the 2,3-bisphosphoglycerate independent phosphorglycerate mutase, are down-regulated in soybean roots under drought exposure [31]. These studies have demonstrated, evidently, that the proteomic characterizations of soybean roots in response to drought stress are important for further investigating the molecular mechanisms regulating the cellular processes in soybean under drought stress. Proteomics methods are generally advantageous in characterizing the response of plants to water scarcity and for further identifying proteins in response to drought stress in soybeans. For example, proteomic studies are capable of detecting the trivial but biologically important changes in the expressions of proteins coping with the drought stress in soybeans [32]. Furthermore, in the present “omics” era, proteomics has gained high popularity over other genome-based technologies due to its ability to explicitly deal with the functional and translational products of the genome. Moreover, proteomics is also a powerful tool for the identification of novel proteins by following both the spatial and temporal changes in protein expressions in soybean [32]. To date, the molecular response to drought stress in soybeans have been extensively studied based on the proteomics analysis of two-dimensional gel electrophoresis (2-DE) technology [32,33,34], whereas the applications of isobaric tags for relative and absolute quantification techniques in proteomics studies of soybeans in response to drought stress are sparse [34]. Therefore, it is extremely important to perform proteomics studies in order to identify the proteins involved in the molecular pathways in response to drought stress in soybean.

In the current study, the molecular response to drought stress in roots of the drought-tolerant soybean variety “Jiyu 47” treated with or without PEG6000 was characterized based on the proteomics approach using TMT methodology. The physiological response to drought stress in soybean roots was also investigated based on a group of six physiological parameters, i.e., the content changes in catalase (CAT), malondialdehyde (MDA), peroxidase (POD), proline (PRO), reduced glutathione (GSH), and oxidized glutathione (GSSH). The objectives of this study were (1) to identify the total proteins and the differentially expressed proteins (DEPs) involved in the molecular response of soybean roots to drought stress based on the database of the Clusters of Orthologous Groups (COG) of proteins (http://www.ncbi.nlm.nih.gov/COG/; accessed on 6 August 2020), (2) to validate the protein expression patterns revealed by the proteomic analysis using the quantitative real-time PCR (qRT-PCR), and (3) to reveal the variations in the metabolic pathways involved in the molecular regulations of the response to drought stress of soybean roots at the translational level based on both the Gene Ontology Annotation (GOA) (http://www.ebi.ac.uk/GOA/; accessed on 6 August 2020) database and the enrichment analysis based on the Kyoto Encyclopedia of Genes and Genomes (KEGG) database (https://www.genome.jp/kegg/; accessed on 6 August 2020). The results showed that the soybean plants responded to drought stress by coordinating their protein expressions during the vegetative stage. This study provided novel evidence to support molecular breeding to improve drought tolerance in soybeans. Our findings provided novel insights into the molecular mechanisms regulating the response to drought stress in soybean, further facilitating the genetic improvement of crops.

## 2. Materials and Methods

### 2.1. Soybean Plants and Physiological Variations in Soybean Roots under Drought Stress

The seeds of drought-tolerant soybean variety (*Glycine max* L. ‘Jiyu47’) used in this study were obtained from the Jilin Academy of Agricultural Sciences, Changchun, China. Seeds were surface sterilized for 10 min in 1.0% (*v*/*v*) sodium hypochlorite, rinsed 5 times with sterile water, and germinated on peat moss in the dark for 3 d at 25 °C. Seedlings were grown in 1 L plastic pots filled with the 1/2 Hoagland’s nutrient solution (pH 5.8). The experiments were conducted in a controlled growth chamber with 60% constant relative humidity and a photoperiod cycle of 14 h light (25 °C and light intensity of 300 μmol m^−2^ s^−1^) and 10 h dark (22 °C). The nutrient solution was replaced every other day. Seven-day-old seedlings were transferred to the 1/2 Hoagland’s nutrient solution containing 20% PEG6000 according to the previous studies [35,36]. The root apices (up to 3 cm in length) treated with and without PEG6000 were collected at 24 h, respectively, immediately treated in liquid nitrogen, and then stored in a freezer (−80 °C) for protein isolation. To evaluate the physiological indices and conduct the qRT-PCR analysis, the root apices (up to 3 cm in length) were collected at 0, 6, 12, and 24 h after the treatment of PEG6000. The root tissues were immediately treated with liquid nitrogen and then stored in a freezer (−80 °C) for further analyses of physiological variations and qRT-PCR. The contents of CAT, MDA, POD, PRO, GSH, and GSSH were determined each based on a total of three biological replicates using the kits (Suzhou Grace Biotechnology Co., Ltd., Suzhou, China) to identify their participation in the molecular mechanisms in response to drought stress in soybean roots. Ultra-violet and visible spectrophotometer (T6, Persee Analytics, Inc., Auburn, CA, USA) were used to measure the optical density (OD) values, each based on a total of three biological replicates to determine the contents of these physiological indices.

### 2.2. Extraction, Tandem Mass Tag Labeling, and Identification of Proteins in Soybean Roots

The samples of root apices each of three biological replicates (Figure S1) were ground into powder in liquid nitrogen and then transferred to a 5-mL centrifuge tube, added with 4 mL of lysis buffer (containing 1% TritonX-100, 10 mM dithiothreitol, and 1% protease inhibitor cocktail, including 50 μM PR-619, 3 μM TSA, 50 mM NAM, and 2 mM EDTA), and followed by sonication on ice with a high intensity ultrasonic processor (Scientz-5T, Ningbo Scientz Biotechnology Co., Ltd., Ningbo, China). The sample was added with a similar amount of Tris-saturated phenol and centrifuged for 10 min at 20,000× *g* and 4 °C. The supernatant was collected and mixed with 5-times the amount of 0.1 M ammonium-acetate/methanol, and incubated overnight at −20 °C. Finally, the protein precipitates were washed with methyl-alcohol and acetone and dissolved in 8 M urea. The protein concentration was measured using a BCA kit (Beyotime Biotechnology, Shanghai, China) according to the manufacturer’s instructions. The protein sample was then diluted with 200 mM TEAB (Sigma-Aldrich, Beijing, China), digested with trypsin (1:50, *w*/*w*; Promega, Madison, WI, USA) overnight at 37 °C, and then digested again with trypsin (1:100, *w*/*w*) overnight at 37 °C [37]. The sample volume of protein solution was adjusted with lysis buffer (20% trichloroacetic acid) slowly added, vortexed, sedimented for 2 h at 4 °C, and the protein precipitate was washed thrice with cold acetone. Then, the supernatant was discarded by centrifugation at 4500× *g* for 10 min. The final concentration of the protein solution was adjusted with 5 mM dithiothreitol (Sigma-Aldrich, Beijing, China) at 56 °C for 30 min and alkylated with 11 mM iodoacetamide (Sigma-Aldrich, Beijing, China) in the dark at room temperature for 15 min. The digested peptides were desalted, and vacuum dried using a Strata X C18 SPE column (Phenomenex, Torrance, CA, USA), then dissolved in 0.5 M TEAB and labeled using the TMT kit (Thermo Fisher Scientific, Inc., Waltham, MA, USA) according to the manufacturer’s instructions. The tagged peptides were fractionated using a high pH reverse-phase high performance liquid chromatography (HPLC) column (Agilent 300Extend-C18, 5 µm particles, 4.6 mm × 250 mm). The peptides were separated into 60 fractions over 60 min using acetonitrile with a gradient of 8% to 32% (pH 9.0; Fisher Chemical, Thermo Fisher Scientific, Inc., Waltham, MA, USA). Then, the peptides were combined into 9 fractions and vacuum centrifuged to dry.

### 2.3. LC-MS/MS Analysis and Protein Annotation

The enriched peptides were dissolved in mobile phase A containing 0.1% formic acid and 2% acetonitrile (Sigma-Aldrich, Beijing, China). The elution gradient increased from 6% to 25% in mobile phase B (0.1 formic acid in 90% acetonitrile) over 26 min, 25% to 35% in 9 min, elevating to 80% in 4 min, and then kept at 80% for the final 4 min. All experiments of the liquid chromatography-tandem mass spectrometry (LC-MS/MS) analyses were performed on the EASY-nLC 1000 ultra-performance liquid chromatography (UPLC) system (Thermo Fisher Scientific, Inc., Waltham, MA, USA) with a constant flow rate of 400 nL/min. The peptides were subjected to the nanospray ion source for nanospray ionization (NSI) followed by MS/MS analysis in Q Exactive (Thermo Fisher Scientific, Inc., Waltham, MA, USA), coupled with the UPLC system. Peptides were then selected for MS/MS analysis using NCE setting at 28 with the fragments detected at a resolution of 17,500 in the Orbitrap. A data-dependent procedure alternated between one MS scan followed by 20 MS/MS scans each with 30 s dynamic exclusion. The automatic gain control (AGC) was set at 5E4. The fixed first mass was set as 100 *m*/*z*. The MS/MS data were processed using the Maxquant v.1.5.2.8 (https://www.maxquant.org/; accessed on 6 August 2020). The tandem mass spectra were searched against a total of 74,863 sequences of *Glycine max* (taxonomy ID: 3847) at the Uniprot database (https://www.ebi.ac.uk/; accessed on 6 August 2020). Trypsin/P was specified as the cleavage enzyme allowing up to two missing cleavages. The mass tolerance for precursor ions was set as 10 ppm in the first search and 5 ppm in the main search, with the mass tolerance for fragment ions set as 0.02 Da. The peptide segments were normalized first by (1) the horizontal normalization, i.e., the quantitative number of peptides in each sample was divided by the mean value of each row, and (2) the longitudinal normalization, i.e., the quantitative value of the peptides in each sample was divided by the median value of all quantitative values in the sample, and the quantitative value of the proteins were extracted from the peptides, i.e., the median value of all peptides was extracted and compared as the quantitative value of the protein. In the computer search, the peptide abundance 0 was replaced with a null value and the null values were not processed. The two-tailed Fisher’s exact test was performed for the identification of significantly modulated proteins. The identification of differentially up-regulated and down-regulated proteins were based on |log2(Fold change)| > 1.3 (*p* < 0.05) and |log2(Fold change)| < 0.77 (*p* < 0.05), respectively. The DEPs were divided into four groups (Q1 to Q4) based on their differentially expressed multiples and the cluster analysis was conducted to identify the correlation of protein functions with different expression multiples (i.e., less than 0.667 in Q1, ranging from 0.667 to 0.769 in Q2, ranging from 1.3 to 1.5 in Q3, and larger than 1.5 in Q4). The false discovery rate (FDR) of the TMT-6plex, protein identification, and the peptide spectrum match (PSM) identification was set to 1%.

### 2.4. Functional Enrichment Analysis of Proteins Identified in Soybean Roots

The Gene Ontology (GO) annotations of the proteome of soybean roots were derived from the UniProtKB (https://www.uniprot.org/help/uniprotkb/; accessed on 6 August 2020) at the Gene Ontology Annotation (GOA) (http://www.ebi.ac.uk/GOA/; accessed on 6 August 2020) database. The identified protein IDs were converted to UniProt IDs and then annotated to GO IDs. The identified proteins not annotated by the UniProt-GOA database were further annotated for GO functional analysis by the InterPro (http://www.ebi.ac.uk/interpro/; accessed on 6 August 2020) database based on protein sequence alignment and the proteins were classified into three categories (i.e., biological process, cellular component, and molecular function) based on the GO annotations. The protein domains and functional locations were annotated based on the InterPro protein domain database (http://www.ebi.ac.uk/interpro/; accessed on 6 August 2020). The proteins were also classified based on the database of the Clusters of Orthologous Groups (COG) of proteins (http://www.ncbi.nlm.nih.gov/COG/; accessed on 6 August 2020). The metabolic pathways were annotated based on the Kyoto Encyclopedia of Genes and Genomes (KEGG) database (https://www.genome.jp/kegg/; accessed on 6 August 2020). Specifically, the KEGG automatic annotation server KAAS (http://www.genome.jp/kaas-bin/kaas_main/; accessed on 6 August 2020) was used to annotate the KEGG database descriptions of the proteins. The annotations were then mapped onto the KEGG pathway database using the KEGG online service tool KEGG mapper. The WoLF PSORT was used to predict the protein subcellular localizations (http://www.genscript.com/psort/wolf_psort.html/; accessed on 6 August 2020). The investigations of the subcellular localizations of the proteins were performed due to the variations in gene expression generally realized in different cell types under different developmental stages and physiological conditions, while proteins located at different cellular structures or locations generally play different functions. A two-tailed Fisher’s exact test with the *p*-value normalized to 0.05 was used for each category of the GO, KEGG, and protein domain enrichment analyses of the DEPs against all identified proteins.

### 2.5. Transcriptional Analysis of Differentially Expressed Genes Involved in Glutathione Metabolism

The transcriptional analysis of five differentially expressed genes (DEGs) encoding the DEPs involved in the metabolic pathway of glutathione was performed using qRT-PCR. Primer 3.0 (http://primer3.ut.ee/; accessed on 6 December 2020) was used to design gene specific primers (Table 1). The housekeeping gene *tubulin* of soybean (GenBank Gene ID 100811275) was used as the internal control. The qRT-PCR was performed on a Mx3005P equipment (Stratagene, La Jolla, CA, USA) with the 25-μL reaction system, including 2 μL cDNA template (50–100 ng), 0.5 μL (10 mM) of forward and reverse primers, respectively, 12.5 μL 2× SYBR Premix ExTaq (TaKaRa, Beijing, China), and 9.5 μL double-distilled H_2_O. The procedures of the melting curve analysis of qRT-PCR were as follows: 95 °C for 30 s, followed by 30 cycles of 95 °C for 5 s and 60 °C for 20 s, 95 °C for 60 s, 55 °C for 30 s, and 95 °C for 30 s. The 2^−ΔΔCt^ method was used to calculate the relative expression level of each gene [38]. The significant difference between treatments was determined based on the Statistical investigation Data Processing System [39]. Each experiment was performed with three biological replicates.

## 3. Results

### 3.1. Effect of Drought Stress on the Physiological Changes in Soybean Roots

**Figure 1:**
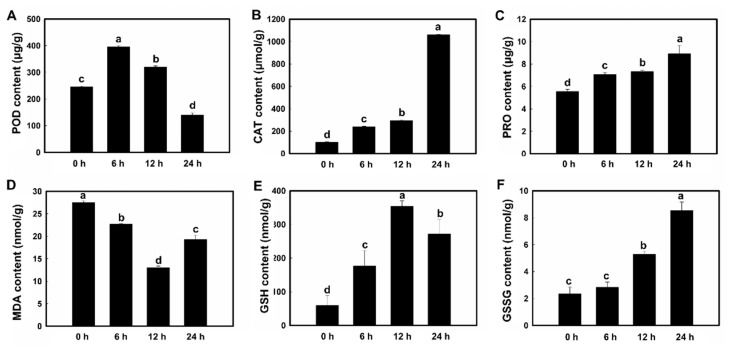
Physiological variations in soybean roots showing the contents of (**A**) peroxidase (POD), (**B**) catalase (CAT), (**C**) proline (PRO), (**D**) malondialdehyde (MDA), (**E**) reduced glutathione (GSH), and (**F**) oxidized glutathione (GSSH). Data are represented as mean ± standard deviation (SD) of three biological replicates. Different letters a, b, c, and d indicate significant differences set at *p* value of 0.05.

In soybean roots, varied patterns were observed in the contents of the six physiological indices. The contents of both PRO and CAT were increased gradually as the treatment time of PEG6000 increased (Figure 1). The contents of POD were significantly increased initially with the treatment of PEG6000, then decreased dramatically in 24 h. The contents of MDA were initially decreased in 12 h and then increased dramatically in 24 h. The contents of GSH were elevated in 12 h, then decreased in 24 h, while the contents of GSSG were increased gradually over the course of 24 h with the treatment of PEG6000.

### 3.2. Protein Profiling of Soybean Roots under Drought Stress

**Figure 2:**
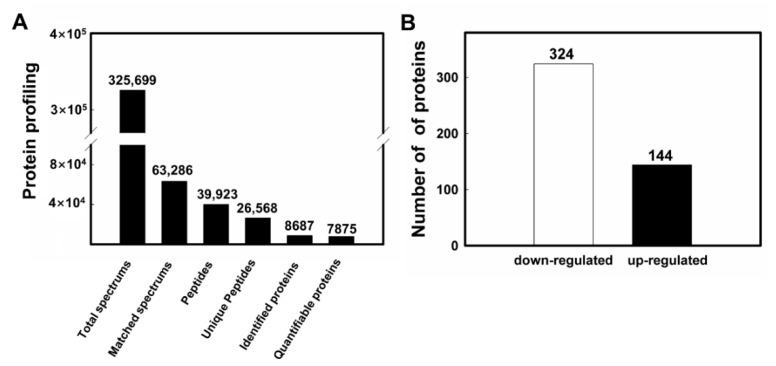
Proteomic profiling of the distributions of the mass spectra (**A**) and the differentially expressed proteins (DEPs) (**B**) induced by drought stress in soybean roots.

Proteomic analysis was performed to analyze the DEPs of soybean roots under drought stress, i.e., with the treatment of PEG6000, in comparison to the soybean roots without the treatment of PEG6000. A total of 63,286 (~19.4%) effective spectra (out of 325,699) were identified in both the control and experimental groups. A total of 26,568 (~66.5%) distinct peptides (out of 39,923) were discovered using the proteomics analysis (Figure 2A). Based on the measurements of the Scaffold Q+ software, a total of 8687 proteins were involved in the response to drought stress with a total of 7875 of these proteins being quantifiable and further analyzed (Table S1). The mass spectrometry proteomics data were deposited to the ProteomeXchange Consortium via the PRIDE partner repository with the dataset identifier PXD033092 (http://www.ebi.ac.uk/pride/; accessed on 7 April 2022). In 24 h, after the treatment of PEG6000, the expression levels of 468 proteins were significantly altered, with 144 up-regulated and 324 down-regulated (Figure 2B).

### 3.3. GO and COG Annotations of Proteins Induced by Drought Stress

**Figure 3:**
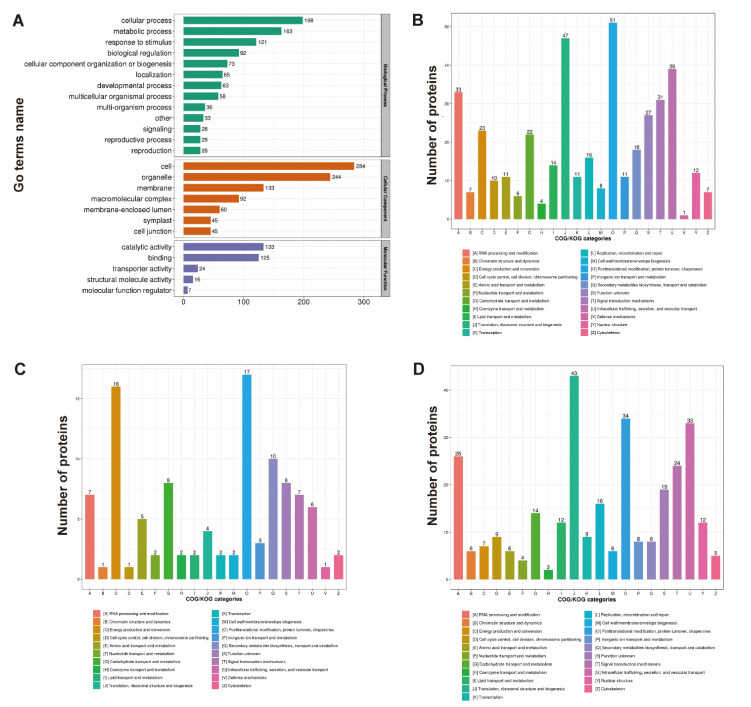
GO annotation of differentially expressed proteins (DEPs) (**A**) and COG functional classifications of total DEPs (**B**), of up-regulated DEPs (**C**), and of down-regulated DEPs (**D**) induced by drought stress in soybean roots.

The proteins induced by drought stress in soybean roots were first enriched based on the GO database. Among the three categories of GO terms, the top five biological processes included the cellular processes (with a total of 198 proteins annotated), the metabolic processes (163 proteins), the response to stimulus (121 proteins), the biological regulation (92 proteins), and the cellular component organization or biogenesis (73 proteins), the three cellular components with the most proteins annotated were cell (284 proteins), organelle (244 proteins), and membrane (133 proteins), while the top two molecular functions enriched with the most proteins were catalytic activity (133 proteins) and binding (125 proteins) (Figure 3A).

In order to further investigate the potential biological functions of the identified proteins in response to drought stress in soybean roots, the protein annotations were then conducted based on the COG database. Results showed that both up-regulated and down-regulated proteins under drought stress for 24 h were enriched into 22 categories based on the COG database (Figure 3B). The top five categories with the most proteins enriched included posttranslational modification, translation, ribosomal structure and modification, intracellular trafficking, secretion, and repair, RNA processing and modification, and the signal transduction mechanisms. The up-regulated proteins were mainly involved in posttranslational modification, secondary metabolites biosynthesis, transport, and catabolism, energy production and conversion, and carbohydrate transport and metabolism (Figure 3C), whereas the down-regulated proteins were mainly enriched in the signal transduction mechanisms, posttranslational modification, intracellular trafficking, secretion, and repair, RNA processing and modification, and the signal transduction mechanisms (Figure 3D). It was noted that the category of the defense mechanisms was only enriched by up-regulated proteins, whereas the nuclear structure was only enriched by down-regulated proteins. Two categories (i.e., the energy production and conversion and the secondary metabolites biosynthesis, transport, and catabolism) were enriched with more up-regulated proteins than the down-regulated proteins, while three categories (i.e., the posttranslational modification, the signal transduction mechanisms, and the intracellular trafficking) were enriched with more down-regulated proteins than the up-regulated proteins. Three categories (i.e., the amino acid transport and metabolism, the nucleotide transport and metabolism, and the coenzyme transport and metabolism) were enriched with the equal numbers of up-regulated and down-regulated proteins.

### 3.4. Subcellular Localization of Proteins Induced by Drought Stress in Soybean Roots

**Figure 4:**
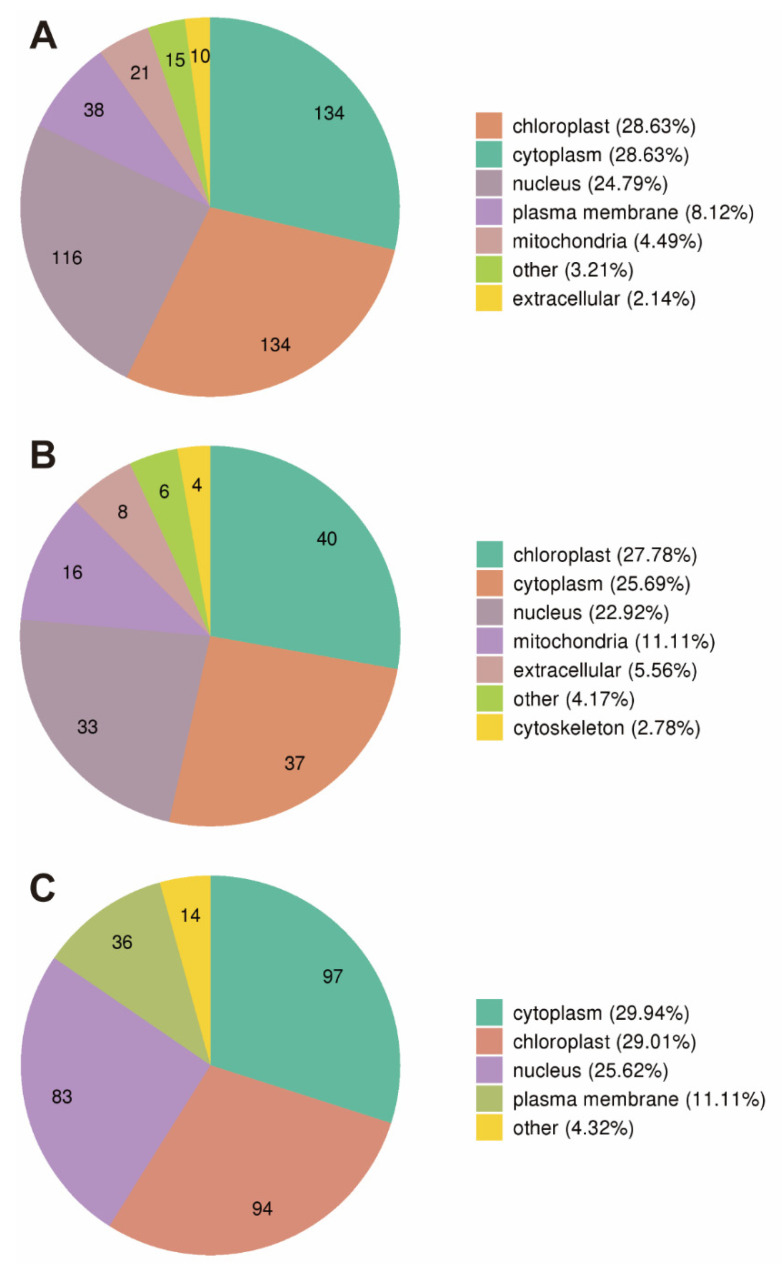
Subcellular localization of the total differentially expressed proteins (DEPs) (**A**), of up-regulated proteins (**B**), and of down-regulated proteins (**C**) induced by drought stress in soybean roots.

The proteins induced by drought stress in soybean roots were mainly localized in the chloroplast (28.63%), cytoplasm (28.63%), and nucleus (24.79%) (Figure 4A). Most of the up-regulated proteins were detected in the chloroplast (27.78%), cytoplasm (25.69%), nucleus (22.92%), and mitochondria (11.11%) (Figure 4B), while the down-regulated proteins were mostly found in the cytoplasm (29.94%), chloroplast (29.01%), nucleus (25.62%), and plasma membrane (11.11%) (Figure 4C). It was noted that all of the plasma membrane proteins, extracellular proteins, or cytoskeleton proteins were up-regulated.

### 3.5. KEGG Functional Annotation and Enrichment Analysis of Differentially Expressed Proteins Induced by Drought Stress in Soybean Roots

**Figure 5:**
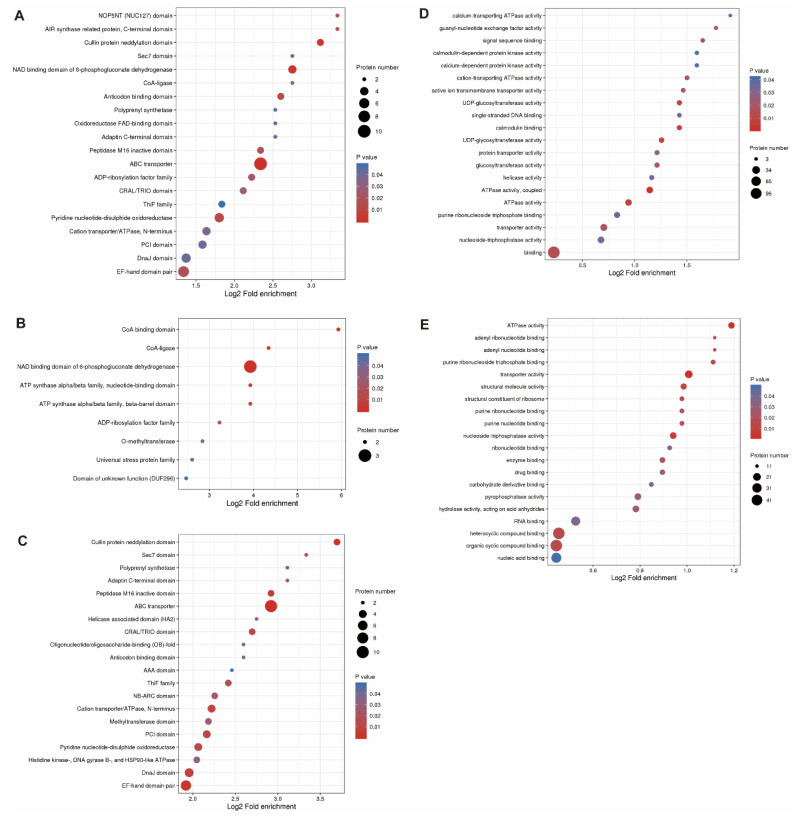
KEGG enrichment analysis of all differentially expressed proteins (DEPs) induced by drought stress in soybean roots (**A**), of up-regulated DEPs (**B**), of down-regulated DEPs (**C**), of up-regulated proteins of molecular functions (**D**), and of down-regulated proteins of molecular functions (**E**).

The InterPro protein domain database was used to identify the substantial enrichment patterns in particular functional classes among the DEPs detected in soybean roots under drought stress. The results showed that the ABC transporter, the NAD binding domain of the 6-phosphogluconate dehydrogenase (6PGD), and the Cullin protein neddylation domain were the most commonly shared functional domains of the DEPs (Figure 5A). The most up-regulated domains included the NAD binding domain of 6PGD and the CoA binding domain, while the ABC transporter, the EF-hand domain pair, and the Cullin protein neddylation domain were the most down-regulated domains (Figure 5B). The most frequently expressed molecular functions of DEPs included the ATPase activity, the calmodulin binding, the UDP-glucosyltransferase activity, and the active ion transmembrane transporter activity (Figure 5C). A total of four molecular functions, i.e., the metal ion binding, the succinate CoA ligase (GDP forming) activity, the 3-hydroxybutyrate dehydrogenase activity, and the UDP-glucose 4-epimerase activity, were enriched with only up-regulated proteins (Figure 5D), while a group of three molecular functions, including the ATPase activity, the transporter activity, and the nucleoside triphosphatase activity, were enriched by only down-regulated proteins (Figure 5E).

### 3.6. KEGG Pathway Enrichment Analysis of Proteins of Groups Q1 to Q4 Induced by Drought Stress in Soybean Roots

**Figure 6:**
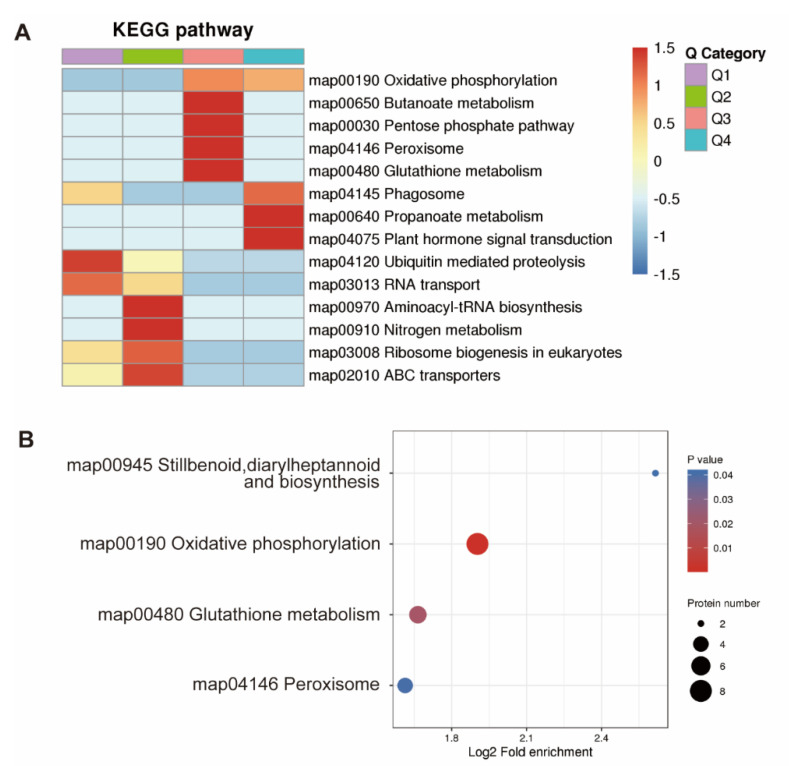
KEGG pathway enrichment of differentially expressed proteins (DEPs) induced by drought stress in soybean roots in clusters of groups Q1 to Q4 (**A**) and in combination of groups Q1 to Q4 (**B**).

Enrichment analysis based on the KEGG database was performed to identify the primary metabolic pathways with the DEPs involved and to investigate the potential biological interactions of proteins (Figure 6). In the Q1 group, proteins were mainly enriched in the ubiquitin mediated proteolysis and RNA transport. In the Q2 group, proteins were mainly enriched in the aminoacyl tRNA biosynthesis, the nitrogen metabolism, the ribosome biogenesis, and the ABC transporters. Proteins were mainly enriched in the butanoate metabolism, the pentose phosphate pathway, the peroxisome and the glutathione metabolism in the Q3 group, while the proteins in the Q4 group were mainly enriched in the phagosome, the propanoate metabolism, and the plant hormone signal transduction (Figure 6A). The diverse groups of proteins were mainly enriched in the oxidative phosphorylation and the glutathione metabolism (Figure 6B).

### 3.7. Glutathione Metabolism in Soybean Roots under Drought Stress

**Figure 7:**
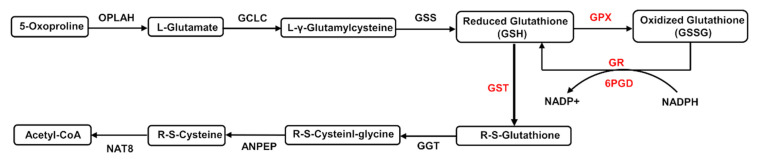
Glutathione metabolism in soybean roots under drought stress. Enzymes showing up-regulation are indicated in red. OPLAH, 5-oxoprolinase (ATP-hydrolysis); GCLC, glutamate-cysteine ligase catalytic unit; GSS, glutathione synthase; GPX, glutathione peroxidase; GR, glutathione reductase; 6PGD, 6-phosphogluconate dehydrogenase; GST, glutathione S-transferase; GGT, gamma-glutamyl transpeptidase; ANPEP, aminopeptidase N; NAT8, *N*-acetyltransferase 8.

The glutathione metabolism in soybean roots is presented in Figure 7. The GSH was oxidized to generate GSSG with the catalysis of GPX. The GSSG was reduced to GSH with the catalysis of GR. The NADP^+^ was catalyzed by 6PGD to generate NADPH and to provide energy for the reaction. The GSH was catalyzed by glutathione S-transferase (GST) to produce R-S-Glutathione, which went through three additional biochemical reactions to generate acetyl-CoA, which served as the intermediate products in other metabolic pathways (i.e., the citric acid cycle and the fatty acid synthesis). Our results showed that the contents of GSH were elevated in 12 h and then decreased in 24 h after the treatment of PEG6000, while the contents of GSSG increased gradually over the course of 24 h after the treatment of PEG6000. These results showed that the expressions of the enzymes GPX, GR, 6PGD, and GST were increased in soybean roots under drought stress, ultimately significantly regulating the generations of the intermediate products in the glutathione biosynthesis.

### 3.8. Validation of the Expression of Differentially Expressed Proteins in Soybean Roots by qRT-PCR

**Figure 8:**
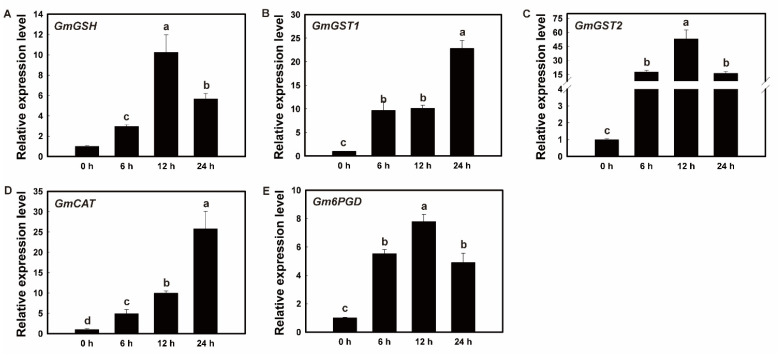
Temporal expression of *GmGSH* (**A**), *GmGST1* (**B**), *GmGST2* (**C**), *GmCAT* (**D**), and *Gm6PGD* (**E**) in soybean roots under drought stress. Data are represented as mean ± standard deviation (SD) of three biological replicates. Different letters a, b, c, and d indicate the significant difference set at *p* value of 0.05.

The qRT-PCR analysis was performed to validate the expression patterns of five genes encoding the GmGSH, GmGST1, GmGST2, GmCAT, and Gm6PGD, which were differentially expressed in soybean roots revealed by proteomic analysis. Selection of these genes was based on their direct involvements in the metabolic pathways closely related to the molecular response of soybean roots to drought stress. In comparison to the control group, similar expression patterns were revealed in genes *GmGSH*, *GmGST2*, and *Gm6PGD*, showing increased expression levels in 12 h and then decreased in 24 h after the treatment of PEG6000, while the expression levels of both genes *GmGST1* and *GmCAT* increased over the course of 24 h of the treatment of PEG6000 (Figure 8). The results of qPCR were consistent with those derived from the proteomics analysis, showing the increased expression of these enzymes, thus validating the protein expression patterns revealed by the proteomics analysis.

## 4. Discussion

### 4.1. Application of Proteomics in the Investigation of Molecular Response to Drought Stress in Soybean Roots

Studies have shown that drought stimulates the expression of drought-resistant genes [40]. Multiple genes involved in distinct metabolic pathways have been identified to influence plant adaptation to drought stress, suggesting that the plant response to drought stress is an extremely complicated process. Comprehensive characterization of the activities of these genes is critical for understanding the molecular processes of crop plants in response to stress [32]. The expressions of these genes induced by drought stress create the molecular networks regulating their unique metabolic and signal transduction pathways. In our study, we explored the molecular mechanisms regulating the metabolic pathways of soybean roots in response to drought stress (i.e., treatment of PEG6000) using the proteomics approach based on TMT methodology.

Traditionally, proteomics analysis is conducted using gel-based and quantitative techniques, as well as cutting-edge MS technology [41,42,43,44,45]. For example, comparative proteomic investigations revealed a total of 107 DEPs in drought-tolerant and drought-sensitive soybean seedlings based on the 2-DE method [46]. In comparison to the 2-DE technique, the proteomics analysis based on the TMT method showed higher sensitivity, lower noise level and increased accuracy and capability of identifying a larger number of DEPs, thus providing novel data for investigations of the molecular mechanisms underlying the response of soybean to drought stress. It was noted that despite the fact that the LC-MS/MS analysis is capable of identifying more proteins, the gel-based techniques are not completely replaced [47,48]. Numerous studies have evidently demonstrated the urgent need of a well-assembled and annotated genome of soybean for protein identification in proteomic investigations, while a novel era of proteomic studies has been significantly facilitated with the completion of the soybean reference genome. For example, a previous study used the same set of LC-MS/MS data generated from the flooding-treated soybean plasma membrane proteome to perform a comparative analysis of the proteins identified, by searching the National Center for Biotechnology Information (NCBI; http://www.ncbi.nlm.nih.gov/; accessed on 24 April 2022) database and the soybean genome sequence [13]. The results showed that a total of 74 proteins were identified based on the NCBI database, while a total of 124 proteins were revealed based on the search of the soybean genome, including 61 proteins discovered from the NCBI database. In our study, the DEPs in soybean roots under drought stress during the seedling stage were investigated using the proteomics analysis with a total of 26,568 distinct (~66.5% out of 39,923) peptides revealed. In comparison to the control group, a total of 8687 DEPs (324 up-regulated and 144 down-regulated) were identified in the soybean roots under drought stress (Figure 2A). These results are consistent with those reported previously, showing that proteomics investigations provided valuable information for improved genomic annotation. For examples, under drought stress, a total of 16 up-regulated and 28 down-regulated proteins were identified in the soybean leaves [49]. These studies provided the candidate proteins involved in the response of soybean to drought stress.

### 4.2. Drought-Induced Variations in the Proteomic Composition of Soybean Roots

Abiotic stresses due to the changes in environmental factors, e.g., temperature (hot or cold), water (waterlogging or drought), and nutrition (overnutrition or malnutrition), trigger complex plant responses through a series of molecular events at both the transcriptional and translational levels. Instead of analyzing the impact of a small number of specific genes on the growth and development of plants, analyses of transcriptomics and proteomics have become common tools for identifying the functions of DEGs and DEPs involved in metabolic pathways that are enriched under stressed conditions. For example, a group of DEGs were identified in sorghum in response to abiotic stresses based on transcriptomic investigations [50,51]. Similarly, the differential expression patterns of *OsMADS* genes in rice were revealed by transcriptomic analysis of breeding rice crops during their growth and development under drought stress [52]. These studies demonstrated, evidently, that the transcriptome plays important roles in response to stress during the growth and development of crops. Furthermore, proteomic dynamics are influenced not only at both transcriptional and translational levels, but also at both post-transcriptional and post-translational levels. In comparison to the transcriptomic analysis, the proteomic studies provided further evidence to support the investigation of the molecular response in plants under stress due to their post-translational modifications and subcellular localizations of proteins. For example, a group of DEGs were identified in tea plants in response to drought stresses based on the proteomic investigations [53], while the DEGs were detected based on the proteomic analysis in alfalfa during their growth and development under osmotic stress [54]. These studies demonstrated evidently that the proteomic studies played an important role in the response of crops to stress during their growth and development stage.

Our results of GO annotation of the proteins in soybean roots in response to drought stress revealed that the enriched biological processes were mainly involved in cellular and metabolic activities, while the cells and organelles were densely packed with cellular components, and the majority of proteins showed catalytic activity as one of their molecular activities (Figure 3A). These proteins identified in soybean roots were further annotated based on the COG database in order to determine and categorize their potential biological activities (Figure 3B). In 24 h under the drought stress, both up-regulated and down-regulated proteins were categorized into 22 functional groups of the COG database, with 7 groups enriched with the most proteins and playing important roles in RNA transcription, signal transduction, post-transcriptional modification, and carbohydrate transport (Figure 3C,D). The up-regulated proteins were mostly engaged in energy generation and secondary metabolite biosynthesis (Figure 3C), suggesting that as the intensity of drought stress was increased, the carbohydrates were needed to provide energy and soluble sugars to sustain osmotic stress in plants. These results were consistent with those reported previously. For example, studies of pod-wall proteome in soybean revealed the rapid induction of proteins involved in stress signaling and regulation, protein folding and stabilization, redox homeostasis, cellular energy, and carbon utilization, as well as the down-regulation of both negative regulators of drought stress and protein degradation related proteins [55]. Furthermore, studies have shown that the defensive mechanisms against redox imbalance and protein misfolding and degradation under stress were enhanced, as suggested by the abundance of proteins involved in redox balance and protein synthesis, assembly, and degradation in soybean at the vegetative stage [56]. Moreover, the coordinated expression patterns of proteins involved in multiple cellular metabolisms, including photosynthesis, oxidative stress defense, respiration, metabolism process, signal transduction, phosphorus transduction, and methyl transduction, were revealed in soybean with the identification of 20 DEPs, based on MS analysis to enhance plant tolerance under drought conditions [33]. Our results showed that both up-regulated and down-regulated proteins were found to be involved in RNA processing and modification, while the down-regulated proteins were mostly involved in translation, ribosomal structure, biogenesis, intracellular trafficking, secretion, repair, posttranslational modification, and signal transduction processes (Figure 3D), whereas almost equal numbers of up-regulated and down-regulated proteins were engaged in amino acid transport and metabolism, nucleotide transport and metabolism, and coenzyme transport and metabolism. It was noted that proteins involved in defense mechanisms were exclusively up-regulated, suggesting that these up-regulated defense proteins were required for soybean to develop drought tolerance or resistance. These results are consistent with those reported previously [33], indicating that the proteomic analysis was useful and important for identifying the metabolic pathways with DEPs involved in soybean roots in response to drought stress, based on COG categorization.

Our results of the subcellular localization analysis of the proteins identified in soybean roots in response to drought stress showed that most of these proteins were found in the chloroplast (28.63%), cytoplasm (28.63%), and nucleus (24.79%) (Figure 4A), with the up-regulated proteins mainly located in the chloroplast (27.78%), cytoplasm (25.69%), nucleus (22.92%), and mitochondria (11.11%) (Figure 4B), and the down-regulated proteins in the cytoplasm (29.94%), chloroplast (29.01%), nucleus (25.62%), and plasma membrane (11.11%) (Figure 4C). It was noted that the plasma membrane proteins, extracellular proteins, and cytoskeleton proteins were exclusively up-regulated (Figure 4). These findings implied that drought stress showed a strong impact on the expression of various organelle proteins in the intracellular membrane-bounded organelles and nucleus. These results were consistent with those reported previously. For example, studies have reported that three genes (i.e., *DREB2*, *AREB*, and *NAC1*) encoding the proteins located in the nucleus played important regulatory roles in signal transduction or transcriptional regulation in tobacco under stressed conditions [57]. Furthermore, studies have shown that the proteins located in chloroplasts participated in the photosynthetic system to enhance the drought resistance in cassava [58], while the membrane transporters were involved in the carbohydrate transport to ultimately facilitate the adaptation of plants under abiotic stresses [59].

The main domains of the various proteins induced in response to drought stress in soybean roots were identified based on the InterPro protein domain database with the goal to reveal the substantially enriched functional domains among these DEPs. The results showed that the ABC transporter, the NAD binding domain of 6PGD, and the Cullin protein neddylation domain were the most commonly shared domains of the DEPs (Figure 5A). The most up-regulated domains included the NAD binding domain of 6PGD and the CoA binding domain, while the ABC transporter, the EF-hand domain pair, and the Cullin protein neddylation domain were the most down-regulated domains (Figure 5B). The most frequently expressed molecular functions of DEPs included the ATPase activity, the calmodulin binding, the UDP-glucosyltransferase activity, and the active ion transmembrane transporter activity (Figure 5C). A total of four molecular functions, i.e., metal ion binding, succinate CoA ligase (GDP forming) activity, 3-hydroxybutyrate dehydrogenase activity, and UDP-glucose 4-epimerase (UGE) activity, were enriched with only up-regulated proteins (Figure 5D), while a group of three molecular functions, including ATPase activity, transporter activity, and nucleoside triphosphatase activity, were enriched by only down-regulated proteins (Figure 5E). These results are consistent with those reported previously. For example, studies have shown that the ABC transporter proteins play a vital role in regulating stress response mechanisms by facilitating the movement of a variety of molecules and ions through the plasma membrane in order to maintain fundamental cellular processes such as ion homeostasis, osmotic adjustment, signal transduction, and detoxification [59], while the UGE was revealed to catalyze the reversible conversion of UDP-glucose to UDP-galactose, with BrUGE1 improving rice growth performance and drought tolerance under optimal and water deficit conditions [60].

### 4.3. Carbohydrate Metabolism in Soybean Roots in Response to Drought Stress

**Figure 9:**
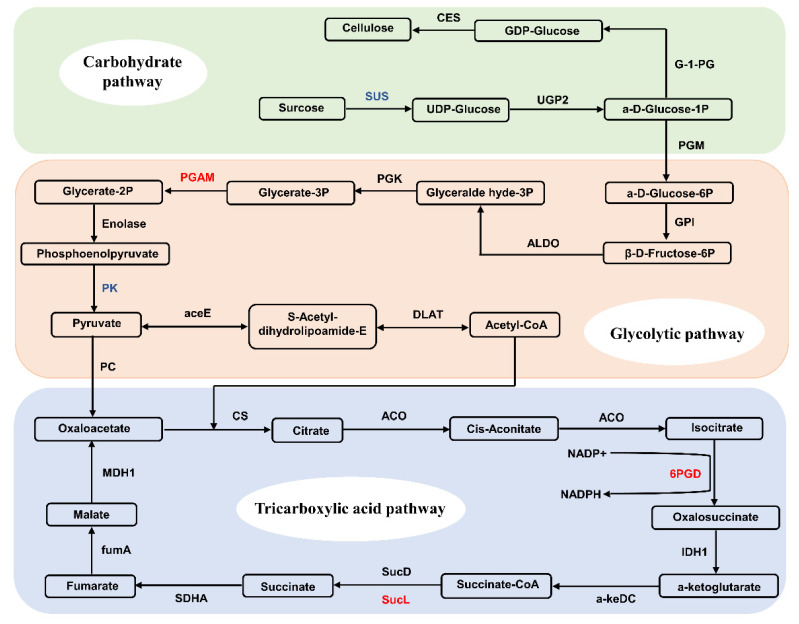
Three metabolic pathways (i.e., carbohydrate pathway, glycolytic pathway, and tricarboxylic acid pathway) in soybean roots under drought stress. Enzymes showing up-regulation and down-regulation are indicated in red and blue, respectively. CES, cellulose synthase; G-1-PG, glucose-1-phosphate guanylyltransferase; SUS, surcose synthase; UGP2, UTP-glucose-1-phosphate uridylyltransferase; PGM, phosphoglucomutase; GPI, glucose-6-phosphate isomerase; ALDO, fructose-bisphosphate aldolase; PGK, phosphoglycerate kinase; PGAM, 2,3-bisphosphoglycerate-dependent phosphoglycerate mutase; PK, pyruvate kinase; aceE, pyruvate dehydrogenase E1; DLAT, dihydrolipoamide acetyltransferase; PC, pyruvate carboxylase; MDH1, malate dehydrogenase; fumA, fumarate hydratase; SDHA, succinate dehydrogenase (ubiquinone) flavoprotein; sucD, succinyl-CoA synthetase; sucL, succinyl-CoA ligase; a-keDC, a-ketoglutarate dehydrogenase complex; IDH1, isocitrate dehydrogenase; 6PGD, 6-phosphogluconate dehydrogenase; ACO, aconitate hydratase; CS, citrate synthase.

Carbohydrate metabolisms in plants are generally regulated by signaling molecules, interacting with other chemicals in the signaling networks. Studies have shown that the carbohydrate metabolisms contribute to the enhancement of drought-tolerance in alfalfa [61]. Furthermore, studies have shown that the decrease in the contents of carbohydrates in plants was associated with the increased level of water stress [62]. Our results showed that drought stress altered the abundance of proteins related to the carbohydrate biosynthetic process, i.e., ribulose-phosphate 3-epimerase, UDP-glucose 4-epimerase, beta-glucuronosyltransferase, succinate-CoA ligase, and sucrose synthase (Figure 9). It is well known that succinate CoA participates in a variety of biological processes, including nucleotide metabolism, carbohydrate derivative metabolism, metal ion response, the generation of precursor metabolites and energy, cadmium ion response, the citrate metabolism, the tricarboxylic acid (TCA) cycle, acyl-CoA metabolism, succinate metabolism, phosphorus metabolism, and the oxidation-reduction process (Figure 9). Our results showed that succinate CoA was involved in the propanoate metabolism and the TCA cycle in soybean roots under drought stress (Figure 5), while the accelerated glycolysis caused an increased level of acetyl-CoA in the TCA cycle, resulting in the generation of a large amount of ATP in response to drought stress. Our results showed that the expression of the sucrose synthase was decreased in soybean roots under drought stress, suggesting that starch and sucrose metabolisms played important roles in the response of soybean to water deficit. Future investigations are needed to explore the molecular mechanisms regulating both the starch and sucrose metabolisms. These results were consistent with those reported previously in the leaves and seeds of soybean under drought stress [63]. Furthermore, the UDP-glucose 4-epimerase was revealed to participate in the galactose metabolism to improve drought resistance in alfalfa [4]. Our study revealed three copies of the UDP-glucose 4-epimerase (2 up-regulated and 1 down-regulated) involved in the galactose metabolism in soybean roots, suggesting that these copies of UDP-glucose 4-epimerase may perform different functions in response to drought stress in soybean. These results indicated that the enhancement of the carbohydrate metabolism contributed to the increased energy production in the cell, ultimately improving drought tolerance in soybean roots under osmotic stress. It was noted that the adaptive mechanisms regulating the maintenance of energy homeostasis between plant growth and defense may vary with different soybean varieties.

### 4.4. Metabolism of the Osmotic Regulation Substances in Soybean Roots

Under drought stress, plants generate a family of highly soluble osmotic compounds with small molecular weights to control cellular osmotic potential, while the resistance to drought stress is generally induced by an increase in the amount of osmosis regulating chemicals, such as carbohydrates, which improve water retention and maintain cell growth under stressed conditions [10]. Furthermore, studies have shown that sugar performs as a type of osmoprotectant by maintaining the osmotic balance, stabilizing macromolecules, and providing a direct source of energy for plant growth [64]. Our results showed that the contents of proline were increased in the soybean roots under drought stress (Figure 1C). These results were consistent with those reported previously. For example, studies have shown that many species of plants accumulate free proline as a type of osmoprotectant in response to environmental stresses [65]. Acting as a type of signal molecule and energy material under stress, proline is shown to not only regulate osmotic pressure, but also strengthen the subcellular structure, as well as scavenge free radicals [66]. Furthermore, under drought stress, proline is mostly synthesized and accumulated in the glutamate pathway with the participation of proline synthase Δ1-Pyrroline-5-carboxylate synthase (P5CS), which is the primary enzyme catalyzing the phosphorylation of glutamate and the reduction in glutamate-1-semialdehyde [67,68]. Studies have shown that the production of proline in transgenic tobacco with the gene encoding P5CS is significantly enhanced under drought stress [69]. Additionally, our results showed that the expression of caffeoyl-CoA-*O*-methyltransferase was increased in soybean roots under drought stress. Studies have shown that the caffeoyl-CoA-*O*-methyltransferase was involved in lignification, causing a reduction in lignin content as an adaptive response to osmotic stress in soybean root [20]. Future studies are necessary to verify the explicit participation and functions of caffeoyl-CoA-*O*-methyltransferase in the response of soybean roots to drought stress.

### 4.5. Enhanced Drought Tolerance by the Antioxidant Defense System in Soybean Roots

Studies have shown that drought stress induces the expression of genes involved in ROS scavenging to activate various drought-related pathways to ultimately induce drought tolerance in plants [70]. In general, plant cells generate a variety of ROS to cause oxidative damage in plants under drought stress, while plants use their antioxidant defense system, i.e., antioxidant compounds and enzymes, for scavenging ROS to enhance their physiological protections in response to drought. Furthermore, the expressions of genes encoding these antioxidants and enzymes are increased significantly under drought stress, demonstrating that the molecular mechanisms of alleviating the effects of drought stress on plants are regulated by the accumulation of antioxidant compounds [71]. Studies have shown that among the known anti-oxidative strategies in plants, the antioxidant enzymes are the most efficient in response to anti-oxidative stress [8]. Plants use numerous antioxidant enzyme systems to eliminate ROS, including SOD, POD, CAT, GST, thioredoxin peroxidase (TPX), and GPX. In addition to both CAT and POD, plants generally maintain high levels of activities for other enzymes under drought stress to eliminate ROS from the cells. The results of our proteomics analysis revealed that a group of DEPs were involved in the glutathione system, while the GSH generated in the glutathione system was catalyzed by a series of enzymes (e.g., GST1) to produce acetyl CoA, which was involved in the TCA cycle. The metabolic products generated in the TCA cycle are generally the materials used in various metabolic pathways, including glucose metabolism, lipid metabolism, and amino acid metabolism; in particular, both sucrose and proline improve drought resistance. Previous studies showed that under drought stress, the transformation of *MruGSTU39* in *Medicago ruthenica* and *M. sativa* enhanced the growth and survival of transgenic seedlings in comparison to their wild-type counterparts [72]. It was further demonstrated that MruGSTU39 detoxified the ROS to reduce its damage to the membrane by up-regulating the activities of both GST and GPX [72]. Furthermore, a *GmGST* gene was revealed to be responsive to drought stress in soybean [73]. Moreover, the overexpression of *CsGSTU8* in *Arabidopsis* resulted in enhanced drought tolerance, as indicated by the improved scavenging of excess amounts of ROS under drought conditions [74]. The results of our proteomics analysis showed that the expression of both GST proteins (i.e., GST1 and GST2) in the GSH system was up-regulated. These expression patterns of both proteins were also validated by the qRT-PCR at the transcriptional level; in particular, the highest expressions were observed in 24 and 12 h for GST1 and GST2, respectively (Figure 8B,C). These results suggest that proteins GST1 and GST2 play different roles in the glutathione system. Further investigations are needed to identify the explicit functions of these two proteins in the molecular response of soybean to drought stress. Furthermore, the contents of PRO in soybean roots were increased gradually as the treatment time of PEG6000 increased (Figure 1D), while the contents of MDA were decreased in 12 h (Figure 1C). These results suggested that both PRO and MDA were rapidly involved in the response to drought stress in 12 h after the treatment of PEG6000 via different mechanisms.

Generally, the GSH/GSSG system removes ROS by regulating the enzymes involved in ROS clearance to enhance antioxidant activities [75]. The GSH participates in ROS scavenging reaction directly by modulating the activity of associated enzymes in the antioxidant enzyme system, while the generation of GSH is primarily controlled by both GPX and GST (Figure 7). Studies have shown that as a member of the peroxidase (POX) antioxidant enzyme family, the GPX decreases the accumulation of lipids and organic hydroperoxides by removing H_2_O_2_ via GSH [76]. Subsequently, GSH is joined with H_2_O_2_ to generate GSSG and to release H_2_O, which mainly takes place in cytoplasm and mitochondria [77]. Our results revealed that the contents of both CAT and POD were significantly increased in 12 h with the treatment of PEG6000, showing the same trends as those of the contents of the GSH/GSSG system (Figure 1A,B), suggesting that the GSH participated in the ROS scavenging reaction directly by modulating the activity of associated enzymes in the antioxidant enzyme system. Plants have evolved a wide range of protective mechanisms in response to stresses. For example, as one of the most significant plant resistance systems, the complex of GSH/GSSG was extensively involved in the signal transduction and stress tolerance in plants [77,78] with the GSH consumed during the ROS detoxification and metabolism, causing the decrease in the content of GSH/GSSG in plants under stress, ultimately changing the condition of ROS in plants. As a signal complex, the GSH/GSSG also triggers numerous defensive mechanisms in plants [79]. For example, one of the main molecular strategies plants utilize to prevent the damage caused by ROS and to cope with abiotic stress is to increase the amount of GSH in cells to respond to excessive ROS induced by the stressed conditions [80]. Our results showed that the contents of GSH were elevated in 12 h after the treatment of PEG6000 but then decreased dramatically in 24 h, while the contents of GSSG were increased gradually over the course of 24 h (Figure 1E,F). Furthermore, the expression levels of *GmGSH* were elevated 8 times in 12 h in soybean roots under drought stress, showing the same expression pattern as that of the contents of GSH (Figure 1E and Figure 8A). These results suggested that 12 h after treatment of drought stress, the GSH/GSSG system was initiated and required to remove ROS in soybean roots. Moreover, the expression pattern of *Gm6PGD* was consistent with the change in GSH content, suggesting the involvement of Gm*6PGD* in the synthesis of GSH in soybean roots under drought stress (Figure 8E).

It was noted that the expressions of many novel proteins and transcription cofactors identified in our proteomics investigation were significantly changed in the soybean roots under stress (Table S1) and involved in a wide range of metabolic pathways, e.g., the S10_plectin domain-containing protein (I1LGD6) involved in the organic cyclic compound binding, the PMEI domain-containing protein (C6SX36) as pectin methyl esterase inhibitor, improving drought resistance by regulating pectin content on the plant cell wall, beta-glucuronosyltransferase GlcAT14A (I1N499), showing UDP-glycosyltransferase activity, the lipoxygenase domain-containing protein (K7KYV7) involved in the metabolism of linoleic acid ultimately altering drought resistance, serine/threonine protein kinase (K7LZV0) involved in regulating the mechanism of phosphorylation, while two transcription factors (i.e., NAC-type C6T2G5 and CCHC-type A0A0R0LPN2) involved in the regulation of drought resistance. Further studies are necessary to identify the functions of these proteins in the molecular response of soybean to drought stress at proteomics level, in particular their physiological and biochemical functions in the metabolic pathways of glucose, lipid, and amino acid metabolisms.

## 5. Conclusions

In this study, we investigated the variations in protein expressions of soybean roots in response to drought stress. Our results showed that drought stress caused significant alterations in the expression of proteins involved in several processes in soybean roots, including carbohydrate metabolism, the metabolism of the osmotic regulation substances, and the antioxidant defense system (including the glutathione metabolism). Specifically, our results suggested that carbohydrate metabolism contributed to increased energy production in the cell, ultimately improving drought tolerance in soybean roots under osmotic stress, while the increased production of GSH enhanced the prevention of the damage caused by ROS and tolerance to abiotic stress. Moreover, glutathione metabolism played a key role in modifying the antioxidant defense system in response to drought stress in soybean roots. Future investigations are necessary to further verify these expression patterns revealed by the proteomic analysis. Our study demonstrated evidently that soybean plants responded to drought stress by coordinating their protein expression during the vegetative stage, providing novel insights into the molecular mechanisms regulating the response to abiotic stress in plants.

## Figures and Tables

**Table 1:** Primers and their sequences used in the quantitative real-time PCR (qRT-PCR) analysis. Letters “F” and “R” indicate the forward and reverse primers, respectively.

Primer	Sequence
GmGSH	F: TGCAGTGTTCAGCATTCCAC R: CCCTCAACCAATACCACAGTCA
GmGST1	F: CTGTGATCAAGGAGGGCCTG R: CCCTCAACCAATACCACAGTCA
GmGST2	F: TCCACAAGAAAGTTCCAGTGCT R: CCCATACAGCTAACACACACTTC
GmCAT	F:GAGTGCTGGAGGCTTTTTGG R: AGTAACTTCTGGATATCCTTCTCAA
Gm6PGD	F: ACTGATCAACCTGTAGACAAGAAA R: GGCCAGTTCACCCAACTTCA
Tubulin	F: GGAAGGCTTTCTTGCATTGGTA R: AGTGGCATCCTGGTACTGC

## Data Availability

The mass spectrometry proteomics data were deposited to the ProteomeXchange Consortium via the PRIDE partner repository with the dataset identifier PXD033092 (http://www.ebi.ac.uk/pride/; accessed on 7 April 2022).

## Supplementary Materials

The following supporting information can be downloaded at: https://www.mdpi.com/article/10.3390/plants11091173/s1, Figure S1: The principal component analysis (PCA) plot showing the relationships among the biological replicates of the soybean root samples treated with (treatment group) and without (control group) PEG6000; Table S1: List of proteins identified in soybean roots under drought stress.
