# Supplementary material for: Proteomic Investigation of Molecular Mechanisms in Response to PEG-Induced Drought Stress in Soybean Roots
Source: Plants (Basel). 2022 Apr 26;11(9):1173. doi: 10.3390/plants11091173 (PMC9100407; doi:10.3390/plants11091173)
Supplement: Supplementary file 1 [file plants-11-01173-s001.zip › Figure S1.pdf]

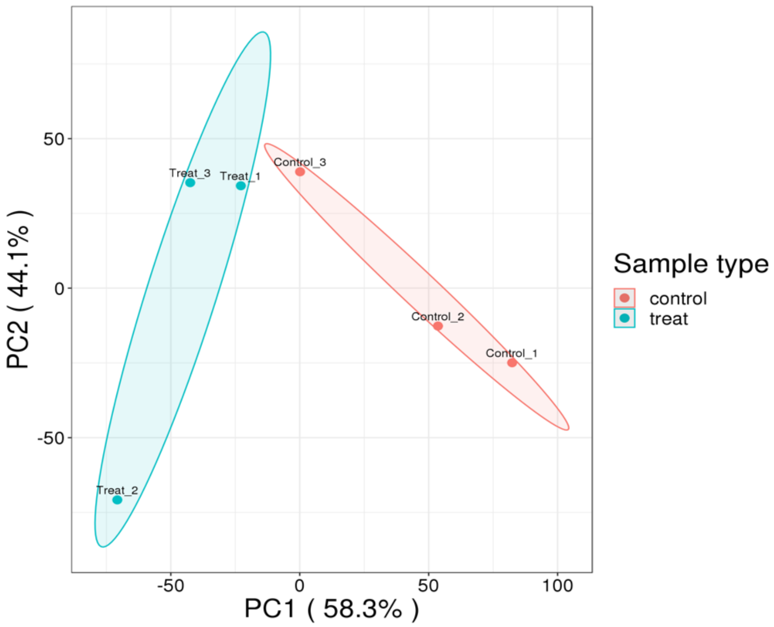

Figure S1. The principal component analysis (PCA) plot showing the relationships among the biological replicates of the soybean root samples treated with (treatment group) and without (control group) PEG6000.
